# Common misconceptions of proper sun protection practices among women wearing hijab in Saudi Arabia: a literature review

**DOI:** 10.1097/JW9.0000000000000263

**Published:** 2026-06-26

**Authors:** Amina Tariq

**Affiliations:** a Department of Dermatology, UMass Chan Medical School, Worcester, Massachusetts, USA

**Keywords:** attitudes, health knowledge, practice, Saudi Arabia, skin cancer prevention, skin neoplasms, sunscreen use, women’s health

**What is known about this subject in regard to women and their families?**

- Saudi Arabia has high ultraviolet radiation exposure but low reported rates of skin cancer.
- Women commonly rely on culturally traditional photoprotective clothing and shade.
- Knowledge of the harmful effects of sun exposure is generally present.
- Sunscreen use among women remains low or improperly practiced, with misconceptions about safety and tolerability.

**What is new from this article as messages for women and their families?**

- There is a measurable gap between awareness of sun damage and correct sunscreen use among women in Saudi Arabia.
- Misunderstanding of sunscreen benefits remains common.
- Cultural clothing practices may currently contribute to lower skin cancer rates.
- As social norms evolve, targeted, culturally sensitive education on sunscreen use is needed to reduce future risk.

## Introduction

Saudi Arabia is one of the most sun-exposed countries in the world with high levels of ultraviolet radiation.^1^ It is also a place with one of the lowest rates of skin cancers worldwide.^2^ Saudi Arabia is a particular area of interest as it is the main holy site of the Islamic world and is a place where traditional protective clothing (such as head scarves and abayas) is worn among women. This study is a review of the knowledge of sun exposure and practices toward preventative measures, such as sunscreen use, in women living in Saudi Arabia. The aim of the study is to determine if there are disparities in knowledge and application of sunscreen use in women living in Saudi Arabia.

## Methods

A comprehensive literature search was conducted using Google Scholar and PubMed databases for studies published between January 2015 and June 2024. The search terms included combinations of “sun exposure,” “sunscreen use,” “sun protection,” “knowledge,” “attitude,” “practice,” “women,” and “Saudi Arabia.”

The inclusion criteria were:

1. Studies conducted in Saudi Arabia;
2. Studies involving female participants;
3. Articles assessing knowledge, attitudes, or practices related to sun exposure or sunscreen use;
4. Studies published in English; and
5. Quantitative or mixed-method designs, including cross-sectional surveys.

Exclusion criteria included studies focusing exclusively on clinical outcomes of skin cancer without behavioral assessment, review articles without primary data, and studies not specific to the Saudi population.

## Results

Our search yielded a total of 7 articles, of which all 7 met the inclusion criteria. Collectively, these papers provided compelling evidence of either low rates of sunscreen use or the improper application of sunscreen use among women. One study conducted a cross-sectional survey of second, third, and fourth year female students on the Sulaymaniyah campus of KAU in Jeddah, Saudi Arabia, showing that there was an overwhelming understanding that sun exposure is harmful to one’s overall health.^3^ However, interestingly, it was seen that 34.1% of women viewed sunscreen as harmful and 64.9% of female students did not know of the protective effects of sunscreen. Interestingly, it was found that the main sun protective method was wearing protective clothing or staying in the shade (78%). This was also seen in the Alsudairy et al.^4^ study, indicating that over 80% would wear long sleeves or head covers to protect from the sun. A common reason cited for avoiding sunscreen use was discomfort with its texture or feel on the skin.^4^

## Discussion

These articles provide compelling evidence that although rates of skin cancer are relatively low in Saudi Arabia, and there is adequate knowledge on the harmful effects of sun exposure, there is clear evidence of either low rates of sunscreen use or improper application of sunscreen use among the majority of women in Saudi Arabia. There is an increased preference for alternative sun protection practices aligned with their culture, such as protective clothing. This is one of the main reasons why there is a low rate of skin cancer in this country despite low rates of sunscreen use. However, it is important to note that with changes in government policies shifting from a conservative to a more liberal perspective in the future years, these factors may play a role in a possible rise of skin cancer cases in Saudi Arabia.^5^ Therefore, it is important to implement programs to educate the population on healthy practices of sunscreen, as well as the importance of sunscreen, and bridge cultural divides and stigma that may be toward sunscreen use in Saudi Arabia. Importantly, future research should aim to better understand women’s preferences, beliefs, and perceptions surrounding sun protection to inform culturally sensitive and patient-centered interventions. Understanding how women perceive different methods of sun protection—whether sunscreen, clothing, or behavioral modification—will be crucial in designing effective public health strategies that respect cultural values while promoting skin health.

## Conflicts of interest

None.

## Funding

None.

## Study approval

N/A.

## Author contributions

AT: Conceptualization, literature review, data collection and analysis, manuscript drafting, critical revision, and final approval of the manuscript.
